# Annotations

**Published:** 1900-04-14

**Authors:** 


### Annotations.

### The Doctor's Pigeons.

The doctor's outfit becomes more extensive and elaborate every year. Time was when with a pocket case, a stethoscope, a few drugs, and a decent nag, he was well set up. But times have changed, and the paraphernalia with which a medical man who makes any pretence to scientific practice must surround himself would considerably surprise our more simple grandfathers. The last straw added, or to be added, to the doctor's burden is the carrier pigeon. In view of the fact that many parts of the country are as yet unprovided with telephones, and that even where these exist it is not every patient who has an installation in his house, the brilliant idea has come into the mind of a writer in the *Gazette des Hôspitaux* that a carrier pigeon left at the patient's house would serve the purpose almost as well, and would enable him to be informed of the progress of the case, or of any evil symptom which might arise. This ingenious gentleman, having trained a number of homing pigeons, takes a few out with him on his rounds, and leaves one or two at the house of any patient in regard to whose progress he wishes to be kept informed, and so well satisfied is he with the working of the plan that he has established a pigeon station in each of the villages around, so that he may be promptly summoned for any case that may require his aid. Thus we find the now familiar war-pigeon arrangement adapted to peaceful purposes. As with the war-pigeon scheme, so with the medical, the entrance to the pigeon-cote is arranged in such a manner that the arrival of the bird rings an electric bell, and we have only to picture to ourselves the unfolding of the missive, and the immediate starting of the long-suffering doctor on the equally obedient and ever ready motor-car, to see how simple and beneficent the whole arrangement is. Perhaps some medicos of ill-regulated minds, not having yet recognised the proper relations between an educated profession and an exacting public, may object to be pigeon-posted all over the country-side in this summary fashion. But, after all, it is not worse than the telephone, and the pigeon with its billet is a far more pleasant messenger than the evil-smelling farmer's man, who sometimes insists on waiting in the doctor's hall until he starts! Moreover, in pigeons there is one great compensation—they will not fly at night.

### Water Gas.

The change which has come over the composition of illuminating gas during recent years is such as cannot but create a certain amount of anxiety in regard to the influence on public health which may be produced by the considerable amount of carbonic oxide which is now so often mixed with it. "Gas," as it is called, has always been more or less poisonous, but only by virtue of one constituent, namely, carbonic oxide, which in old days, when gas was the unadulterated product of the distillation of coal, was present in only small proportions, say about 7 per cent. Nowadays, however, gas companies do not hesitate in an emergency to mix very large quantities of this poisonous compound with their coal gas, and to send it out to their customers without a word of warning, while some companies habitually send out a compound containing over 50 per cent. of carbonic oxide. So far we have not much proof of many deaths having been caused by this compound in England, but in America, where they have a longer experience of its use, the danger has been shown to be very considerable. In a paper on the subject, read by Dr. Haldane before the Society of Medical Officers of Health, he puts the matter in a somewhat striking form when he says that "the total death-rate from poisoning of every kind in this country, whether by gases, liquids, or solids, and whether accidental or suicidal, is only about half the average death-rate from water-gas poisoning alone in Boston, New York, San Francisco and Washington." We do not think, however, that the evil consequences arising from the inhalation of water gas ought to be measured exclusively by deaths; even in non-fatal doses carbonic oxide is definitely deleterious to health, and not improbably, considering the leaky condition of many gas pipes, is the active cause of many mysterious maladies which are only relieved by change of air.

### Milk Standards.

Dr. Harris, medical officer of health for Islington, has recently reported a series of analyses of milk, some of the samples being collected from the churns as they arrive at Finsbury Park Station, and others being taken in the district; and he is able to show that, while the milk as it arrives at Finsbury Park is of good quality, it has been reduced with water and deprived of much of its fat before it reaches the public. But what can one expect when the Somerset House standard is lower even than this adulterated milk? Take the question of butter fat, alias cream. The average of 30 samples taken at Finsbury Park gave a percentage of 3·91 of fat, and the average of 49 samples taken in the district gave a percentage of 3·57, showing that about an eleventh of all the fat present had been removed. Somerset House, however, is willing to acccept 3·0 per cent. of fat as representing pure milk. What, then, is the milk dealer to do? Is he to sell his rich milk as he gets it—*i.e.*, far above the standard—and yet only get standard price for it? or is he to dilute it down to what he knows will pass? Practically, he is forced by competition to do the latter. We venture to express the opinion that the milk trade will never be placed upon a satisfactory footing until two, if not more, standards of milk are recognised, one being, say, that now constantly reached by the milk as it arrives at Finsbury Park Station—namely, fat 3·91 per cent., and total solids 12·76 per cent., or even higher; and the other—the seconds—being of the standard demanded by Somerset House—namely, fat 3·00 per cent., and total solids 11·50 per cent. It is clear that natural milk, even when untampered with is far too variable a substance to be judged of by one standard. If the standard is fixed too high the poor farmer, who sells the honest product of an honest but miserable cow, gets fined unjustly; while if, as at present, it is fixed by the "miserable cow," the splendid milk produced on high-class farms is but diluted down to this unfortunate standard on its way to the consumer. An official recognition of "best" and "seconds" would meet the difficulty, and the dealer could then be forced to sell his milk for what it is.

